# Editorial: Mitochondrial Exchangers and Transporters in Cell Survival and Death

**DOI:** 10.3389/fphys.2021.745353

**Published:** 2021-09-08

**Authors:** Wai-Meng Kwok, Emad Tajkhorshid, Amadou K. S. Camara

**Affiliations:** ^1^Department of Anesthesiology, Medical College of Wisconsin, Milwaukee, WI, United States; ^2^Department of Pharmacology and Toxicology, Medical College of Wisconsin, Milwaukee, WI, United States; ^3^Cardiovascular Center, Medical College of Wisconsin, Milwaukee, WI, United States; ^4^Cancer Center, Medical College of Wisconsin, Milwaukee, WI, United States; ^5^Department of Biochemistry, University of Illinois at Urbana-Champaign, Urbana, IL, United States; ^6^Theoretical and Computational Biophysics Group, NIH Center for Macromolecular Modeling and Bioinformatics, Beckman Institute for Advanced Science and Technology, University of Illinois at Urbana-Champaign, Urbana, IL, United States; ^7^Center for Biophysics and Quantitative Biology, University of Illinois at Urbana-Champaign, Urbana, IL, United States; ^8^Department of Physiology, Medical College of Wisconsin, Milwaukee, WI, United States

**Keywords:** VDAC, mitochondrial Ca^2+^ uniporter, ischemia-reperfusion injury, chloride channels, translocator protein, inflammatory pathways, mitochondria, LETM1

Dysfunction of mitochondria underlies the pathogenesis of a myriad of diseases. One of the main sources of mitochondrial dysfunction is associated with dysregulation of ionic and metabolite fluxes across the mitochondrial membranes. Maintaining ionic homeostasis is key to normal mitochondrial function. Examples include proton (H^+^) pumping by the electron transport chain (ETC) complexes, I, III, and IV, across the inner mitochondrial membrane (IMM), which provides the energy source to produce ATP by reentry of H^+^ via complex V, the ATP synthase. Mitochondria are also a significant hub in regulating cytosolic Ca^2+^ (Rizzuto et al., 2012). Major components involved in mitochondrial Ca^2+^ dynamics that underlie this regulation are the voltage-dependent anion channels (VDACs) in the outer mitochondrial membrane (OMM), the mitochondrial Ca^2+^ uniporter complex (MCUC) in the IMM for Ca^2+^ influx and the Na^+^/Ca^2+^ exchanger (NCLX) in the IMM for Ca^2+^ efflux. As a testament to the physiological significance of ionic homeostasis, the mitochondrial membranes are equipped with a plethora of transporter and ion channel proteins (Szabo and Zoratti, 2014), with the potential for more to be uncovered. The functional significance of some of the proteins discovered has yet to be established. Filling these knowledge gaps will be instrumental in identifying novel therapeutic targets in mitochondria for the treatment and management of a wide spectrum of maladies including cardiovascular, neoplastic, and neurodegenerative diseases.

This Research Topic sheds light on and provides additional insights into the roles of a variety of mitochondrial transporters and ion channels under physiological and pathophysiological conditions. In three original research articles, the functional role of LETM1 (leucine zipper-EF hand containing transmembrane protein 1), the putative mitochondrial Ca^2+^/H^+^ exchanger, CHE (Natarajan et al.), differential impact of the ETC complexes between *in vivo* and *ex vivo* ischemic heart model with implications in heart transplantation (Quader et al.) and a novel use and underlying mechanisms of PK11195, an isoquinoline carboxamide, as a cardioprotective agent against ischemia-reperfusion (IR) injury (Seidlmayer et al.) are addressed. In addition to these illuminating articles, four review articles provide in-depth and unique insights into: (1) the controversy on the functional role of LETM1 (Natarajan et al.), (2) modulation of VDAC1 by steroids and hydrophobic drugs (Rostovtseva et al.), (3) mitochondrial Cl^−^ channels in pathologies and their potential as therapeutic targets (Rao et al.), and (4) roles and significance of mitochondrial ion channels in inflammatory response (Ponnalagu and Singh).

Treatment and management of ischemic heart disease continues to be a considerable challenge. Although reperfusion therapy is the most effective strategy, it can also lead to further injury to cardiomyocytes due to the well-documented cytosolic and mitochondrial Ca^2+^ overload triggered at the onset of reperfusion. Mitochondrial Ca^2+^ overload and ETC dysfunction lead to increased generation of reactive oxygen species (ROS). The 18-kDa translocator protein, TSPO, in the OMM has been reported to increase its expression in response to stress (Thai et al., 2018), as in heart failure; thus, it is potentially a therapeutic target for cardioprotection. However, inhibition of TSPO has resulted in conflicting outcomes, including both protective and damaging effects. In a highly systematic study, Seidlmayer et al. report on a novel cardioprotective outcome of PK11195, an inhibitor of TSPO. It should be noted that PK11195 also inhibits the F-ATP synthase (Cleary et al., 2007). Interestingly, PK11195 proved protective against IR injury only when applied at the onset and during reperfusion. The protective effects of PK11195 included decreased cell death and limited ROS-induced ROS release. Mitochondrial membrane potential was preserved with maintenance of matrix Ca^2+^ during reperfusion coupled with limited succinate oxidation in favor of glutamate oxidation. The underlying molecular mechanism of the protective effects of PK11195 is yet to be established but may include a TSPO-independent component.

In an intriguing study that may have implications in heart transplantation, Quader et al. report on the differential impact of *ex vivo* and *in vivo* global ischemia on mitochondrial function. A major prohibiting factor in heart transplantation is the limited availability of donor hearts, traditionally acquired via donation after brain death (DBD). To potentially increase the pool of available hearts for transplantation, a clinical trial (Shudo et al., 2021) is underway to investigate the use of hearts procured from donation after circulatory death (DCD), as in the case where life support is withdrawn. Insofar as DCD involves a period of ischemia, Quader et al. investigated mitochondrial dysfunction *in vivo* in an animal model of IR injury that mimicked DCD conditions. As numerous animal studies on IR injury utilize the isolated *ex vivo* heart model, they compared the *in vivo* findings with those from the *ex viv*o model. Although common deficiencies in mitochondrial function were observed in both *in vivo* and *ex vivo* models, including impaired Ca^2+^ retention capacity, the degree of damage to mitochondrial function was more pronounced in the *ex vivo* model. This included a greater decrease in protein yield from interfibrillar mitochondria which have been reported to be more resistant to ischemic damage compared to subsarcolemmal mitochondria. The respiratory control ratio was also significantly lower in the *ex vivo* group due to a decrease in state 3 respiration and an increase in state 4 respiration. The observation that the *in vivo* group exhibited less damage compared to the *ex vivo* group could be attributed to the presence of blood in the former that may act as a buffer.

Mitochondrial Ca^2+^ influx and efflux are highly regulated. Dysregulation can exceed the matrix capacity to buffer Ca^2+^ and ultimately result in opening of the mitochondrial permeability transition pore, mPTP (Carraro et al., 2020). The extent to which CHE contributes to mitochondrial Ca^2+^ regulation in the heart is unclear. This is further confounded by the observation that LETM1, the putative molecular identity of CHE, may also act as a K^+^/H^+^ exchanger (KHE). To address the role of CHE, Natarajan et al. report on the impact of matrix Ca^2+^ and pH in regulating Ca^2+^ flux via the CHE, based on a series of experiments complementing pharmacological studies with a LETM1 knock-down cell model. A novel dynamic three-way relationship is proposed whereby activation of CHE is dependent on the availability of matrix or buffered Ca^2+^ to bind to the CHE. In this scheme, Ca^2+^ efflux increases via the CHE when available Ca^2+^ increases, either through impaired matrix Ca^2+^ buffering capacity or an increase in steady-state matrix Ca^2+^. This revealed a unique relationship between CHE activation and the mitochondrial Ca^2+^ handling capacity.

In a related review article, Natarajan et al. discuss the multifaceted roles of LETM1 in health and disease. Evidence for LETM1 as a CHE or KHE is presented. The “mystery” of LETM1 goes beyond its identity as an exchanger since its diverse cellular impacts in the context of pathophysiology are well-documented. Indeed, *Letm1* gene was first identified as one of the deleted genes in the Wolf-Hirschhorn syndrome which is marked by slow growth, intellectual disability and seizures (Nowikovsky et al., 2004). The concise review highlights the critical roles of LETM1 in metabolic dysregulation, neuronal disorders, and cancer.

Mitochondrial VDAC1 is the most abundant protein expressed in the OMM. It is the main gatekeeper for the passage of metabolites, nucleotides and ions in and out of mitochondria and acts as a convergence point for cellular signaling, including those associated with cell survival and death (Camara et al., 2017). Because of the functional importance of VDAC1, it is recognized as an attractive therapeutic target. In fact, the potential of VDAC1 as a target for anti-cancer therapy is well-documented. Recent studies have reported on the ability of a wide variety of steroids and non-polar drugs to bind to VDAC1. This could potentially lead to alterations in mitochondrial function via their impact on VDAC1. In an insightful review by Rostovtseva et al., these drugs are discussed in the context of their effects on VDAC1 channel properties. The review expertly articulates the mechanisms underlying the drug effects. These include direct interaction of the drugs with VDAC1 at the protein-lipid interface or indirectly via modification of lipid bilayer properties surrounding the channel. Additionally, Rostovtseva et al. note that some drugs may not affect basic channel characteristics but alter VDAC1's ability to bind with cytosolic proteins, which would need to be considered in further mechanistic characterization.

A detailed review by Rao et al. discuss a novel class of intracellular organelle ion channels, specifically the chloride intracellular ion channels, CLICs. Chloride has long been known as a major signaling ion involved in cell volume regulation, proliferation and apoptosis. CLICs are the most recent family of Cl^−^ channels discovered and consist of six members, CLIC1-6. One of the key intracellular organelles where CLICs are found localized is mitochondria, and dysfunction attributed to various CLIC channels has been implicated in a multitude of diseases, such as cancer, hearing impairment, and pulmonary hypertension. The review by Rao et al. nicely documents and discusses the role of various members of the CLIC family, including their physiological roles, implications in pathophysiology, and their potential as therapeutic targets. Rao et al. also raise a caveat in that CLICs are dimorphic and can exist both as soluble cytosolic proteins and as ion channel proteins on organelles or in the plasma membrane, which brings an additional layer of complexity when delineating the pathophysiological roles of CLICs.

Mitochondria have emerged as key components in the cellular response to inflammation. For example, dysregulation of mitochondrial Ca^2+^ dynamics can lead to increased ROS production and impact the inflammatory pathways. A comprehensive review by Ponnalaug and Singh provides a highly informative treatise on the role of mitochondria in the inflammatory response. Topics include the regulation of inflammasome formation by mitochondria, significance of damaged mitochondrial DNA in the inflammatory response, and mitochondrial apoptotic signaling. Special emphasis is placed on the role of various mitochondrial ion channels and transporters, such as CLICs, MCUC, and uncoupling proteins, as their involvement in the inflammatory response is not well-understood. Filling the knowledge gap in their roles can provide new mitochondrial targets in the treatment for inflammatory disorders.

Mitochondria are commonly referred to as the powerhouse of the cell, mainly due to their production of the cellular energy currency, ATP. However, it is well-known that mitochondria are also involved in a wide variety of other cellular functions, ranging from regulating cytosolic Ca^2+^ to generating ROS, to playing key roles in cell death and survival pathways. Synergistic relationships among ion channels and transport proteins in mitochondria are paramount to achieving these multi-functional roles. This Research Topic provides a window into the various roles of these mitochondrial membrane proteins in maintaining mitochondrial function and their contributions in pathophysiology. It further highlights the potential for these proteins as novel therapeutic targets in the treatment of a variety of diseases.

## Author Contributions

W-MK, ET, and AKSC contributed to the conception and design of this Research Topic. All authors contributed to and approved the editorial.

## Funding

This work was supported by NIH R01 HL131673.

## Conflict of Interest

The authors declare that the research was conducted in the absence of any commercial or financial relationships that could be construed as a potential conflict of interest.

## Publisher's Note

All claims expressed in this article are solely those of the authors and do not necessarily represent those of their affiliated organizations, or those of the publisher, the editors and the reviewers. Any product that may be evaluated in this article, or claim that may be made by its manufacturer, is not guaranteed or endorsed by the publisher.
